# The Battle of the "Pathies"

**Published:** 1888-01-21

**Authors:** 


### The Hospital

A Weekly Institutional Journal of

**Science, Medicine, and Philanthropy.**

*For Hospitals, Asylums, Medical Practitioners, Students, Nurses, Families, Parishes, Congregations, and the Charitable Public.*

Vol. III. No. 69.] [January 21, 1888

### THE BATTLE OF THE "PATHIES."

The *Times* has opened its columns to Lord Grimthorpe, who uses a sledge-hammer kind of logic in defence of Homœopathy. Dean Ramsay records of a certain Scotchman that, when very angry, he found great relief in swearing; and if he happened to fly into a passion when nobody was within hail whom he could swear at, he promptly put himself into a convenient attitude and "swore at large." Lord Grimthorpe reminds us strongly of that particular Scotchman. He undertakes to discuss a question of which he has neither the knowledge nor the wit to understand the significance; and, knowing that, he straightway takes up the biggest sledge-hammer he can lay hands upon, and, like the swearing Scotchman, beats the air "at large." That is a highly amusing proceeding, and capital fun for the readers of the *Times;* most of all for the doctors, who fully appreciate the ridiculous figure his sapient lordship cuts. We would not hint that he has been specially retained by the *Times* to don the cap and bells for the enlivenment of the dull season. But if he had been, he could not have accomplished the object more successfully.

An argument with Lord Grimthorpe we do not propose to ourselves—at any rate on the particular topic on which he has chosen to amuse the public through the columns of the *Times.* An argument pre-supposes some equality of knowledge and some compatibility of aim and temper. The schoolmaster does not argue with the refractory urchin, who makes a new sensation for himself by tying a lighted squib to the tail of the matron's cat; he birches him.

But whilst Lord Grimthorpe and his showman's talents may be valued at what they are worth by ourselves and other medical men, they will not perhaps be so valued by outsiders. Because it is *Lord* Grimthorpe who speaks, many people will listen; and the foolish among them will store up the words in their memories for foolish future use. In defending homœopathy Lord Grimthorpe tries to show that medical men as a body may very properly admit to each other, and of course therefore to the world, that "the dramatists and satirists of all ages have been right in representing us as humbugs." That is a charge which may very well be left to answer itself. It is a settled rule with the writer of this article, when he hears a person say he does not believe there is a really honest lawyer in the world, to consider that person a knave; and so also when any man by implication denounces a whole class of his fellow-countrymen, most of them of equal, and many of them of superior education and proved ability to himself, as "humbugs," that man is at once placed, by the same rule, in the very class and under the very label which he is so anxious to affix upon others. We do not say Lord Grimthorpe is a "humbug." The readers of his letters in the *Times* can think and say what they please.

What we are concerned to do now is, not to try conclusions with any outsider, or to decide the question of the value and position of homœopathy. Sectarianism in medicine, like sectarianism in religion, is out of date. It is the offspring of narrowness of mind and deficiency of knowledge. Homœopaths are distrusted by other practitioners because of their voluntary and ostentatious separatism—a separatism which has no foundation either in necessity or reason. The medical man of philosophic mind and honest purpose knows nothing whatever about "isms" and "pathies," about great names and famous schools, in his daily practice. What he does know is, that people in sickness entrust their lives to his care, and that he is bound by every consideration of humanity, of honour, of reason, and of religion to render them the utmost possible aid which his knowledge, experience, and special training dictate until they die or are restored to health. That, we make bold to affirm, is the main principle by which every medical man of average character is constantly guided, and that is the principle which we hope and believe underlies the conduct of every other decent Englishman in whatever calling he may be engaged. We do not for one moment believe in the pessimist's howl that all men are knaves and liars. Let the pessimist wash his own skin before he ventures to declare his neighbour black.

In carrying out the great principle of always doing the utmost that lies in his power for his patient, the right-minded medical man does not hesitate to use any method whatsoever which he has reason to believe will be of service. Orthodox medicine gives a man the fullest liberty to practice in any way he pleases, provided his methods are such as can be justified by results. Once and again when the present writer has been asked if he objected to a given drug because it was a "homœopathic" preparation, has he declared that he objected to nothing in heaven or earth that had ever been known to do any good, or that could by any possibility be supposed capable of doing any good. "Extract of saddles and bridles," "infusion of chair legs," "decoction of church steeples"—anything and everything he has affirmed his willingness to try, if there seemed the slightest possibility of its being of real service to the patient.

That, we boldly maintain, is the attitude of the "scientific medicine" of to-day; and we use the words "scientific medicine" with the utmost confidence and openness. There is a "scientific medicine," a medicine which, whatever its failures—and they are many—returns, and again returns, to the arduous conflict in which it is engaged. Who has penetrated most profoundly the secrets of Nature? Who has striven, under showers of unmerited obloquy, to find out the meaning of all kinds of disease, and to discover or create remedies for its cure? Who has made every variety of dangerous experiment upon himself, not only at the risk but often with the loss of his own life? Who, we ask, but the doctor?

We admit to the full that medicine is not in every department an exact science, but a more or less successful art. And though we readily admit the want of exactitude, we do not admit it with a blush, but with a sigh. Honest doctors invariably give their best to their patients—and a highly-skilled and instructed best we affirm it to be. What can any mortal man do more? If the public desire more exactitude, more power over disease, more success in prolonging life, so also do medical men desire these things, and with an urgency infinitely greater. Whatever they may have been in the past, medicine and surgery are now honest, intelligent, skilled, and successful arts, and in certain departments are as precise and exact as any other science whatsoever. If it were necessary to prove this, we have only to appeal to the experience of every man and woman who has been inside a hospital, of almost every head of a family in the civilised world, of every missionary in savage lands, and of every sick soldier who has known the dangers of the battlefield.

But it is not necessary to prove so self-evident a proposition. There is no class of men who are more trusted and more beloved than the doctors of the present day; none who can look the world more openly in the face and say with truth: "We have come to you in your hour of need; we have given up rest and sleep; we have thought and toiled that you might have the best both of heart and brain. You in turn have given us, not merely your money, but that which is above all price, your friendship, your affection, your trust." No medical man in the kingdom, who has acted honestly and kindly by his patients, need fear the effect of Lord Grimthorpe's words, even though they should be published in every newspaper in the world. A life well spent in good work well done is the surest of all defences when the day of conflict dawns.

